# Patterns of Use and Knowledge about Contact Lens Wear amongst Teenagers in Rural Areas in Malaysia

**DOI:** 10.3390/ijerph16245161

**Published:** 2019-12-17

**Authors:** Bariah Mohd-Ali, Xuan Li Tan

**Affiliations:** 1 Optometry and Vision Science Program, Faculty of Health Sciences, Universiti Kebangsaan Malaysia, Kuala Lumpur 50300, Malaysia; 2 Faculty of Optometry and Vision Sciences, SEGI University, Petaling Jaya, Selangor 47810, Malaysia; tanxuanli@segi.edu.my

**Keywords:** contact lens, teenagers, knowledge, prevalence, rural

## Abstract

Background: Contact lenses (CLs) are more popular than spectacles for vision correction amongst the youth. Knowledge about the risks of wearing CLs is critical especially for those with poor access to public health education. This study investigates the patterns of use and level of knowledge about CL wear amongst teenagers living in rural areas in Selangor, Malaysia using a set of validated questionnaires. Methods: A total of 8500 self-administered questionnaires were distributed in eight selected secondary schools. The results were analysed using descriptive statistics. Results: A total of 2474 (29%) completed questionnaires were collected. The mean age of the respondents was 14.8 ± 1.5 years, and approximately 7.2% were CL wearers. The majority of the wearers were females (76.0%) and wore soft CLs (92.2%). Cosmetic purposes (58.1%) and comfort (24.6%) were the main reasons for wearing CLs. Many of the respondents purchased their lenses from optical shops (50.1%) and beauty accessory shops (15.6%), and approximately 10% did not disinfect their lenses properly. Regarding knowledge about CL care, approximately 56% of the respondents responded correctly. Conclusion: Half of the respondents do not have sufficient knowledge about the risks of wearing CLs. Thus, aggressive public health education aimed at teenagers is needed to prevent improper CL usage.

## 1. Introduction

Advances in the field of contact lenses (CLs) have allowed for safe and practical CL wear amongst the pediatric population. With improved materials for oxygen permeability in lenses, children can wear CLs for prolonged periods, which helps enhance the children’s physical activities and social acceptance [1]. Using a survey on the pediatric quality of life, Walline et al. [2] and Rah et al. [3] have demonstrated how CL wear improves the perception of children (8–12 years) and teenagers (13–17 years) on their appearance and participation in activities, which leads to an enhanced satisfaction with their refractive error correction. Compared with spectacles, wearing CLs improves visual acuity (due to increased magnification in myopia), provides a wider field of view and eliminates prismatic peripheral distortion [4]. 

Nevertheless, a number of eye care practitioners are reluctant to prescribe CLs to children due to safety issues. The success of CL wear has often been threatened by incidents of adverse events, such as microbial keratitis. Improper behavior, such as poor cleaning, disinfection using reused solutions and lack of case replacements, have been suggested as reasons for complications and CL failures [5,6]. Apart from these behaviors, poor understanding of proper hygiene and CL care is also an issue. According to de Oliviera et al., 30% of the respondents in a 2003 survey considered themselves poorly prepared for CL care and maintenance, suggesting a lack of knowledge amongst the wearers [7]. Failure to adhere to instructions given by optometrists is another area of non-compliance that has been repeatedly identified. In another survey, CL non-compliance has been found to be 98% with problematic activities arising in the following order (most common first): tap water exposure, sleeping in unapproved lenses, overwear, failure to wash hands, delayed lens case replacement and solution misuse [8]. 

In 2016, an estimated 3.6 million adolescents aged 12–17 years (14.5% of adolescents), 7.5 million young adults aged 18–24 years (24.4% of young adults) and 33.9 million adults aged >25 years (15.5% of adults) in the United States wore CLs [9]. The estimation showed an increased prevalence of CL wear amongst young adolescents, which was probably due to the increased variety of CLs, easy over-the-counter or internet purchases, and cheaper costs compared to those in the past. In 2009, optometrists in Malaysia were projected to have prescribed approximately 90 new pairs of CL a year [10]. Nevertheless, we believe that the numbers are now growing due to the increasing prevalence of myopia and the use of orthokeratology CLs in myopic school children [11,12]. To our knowledge, no data are available in the literature about the patterns of CL wear amongst teenagers in rural areas in Malaysia. In a previous survey of CL prescriptions in Malaysia, Mohidin et al. showed that approximately 67% of CLs were prescribed annually to patients aged approximately 21 years [10]. Nevertheless, CLs have become a popular option for teenagers to correct refractive error with the increasing prevalence of myopia in Malaysian schoolchildren [13]. Thus, teenagers should be informed of the risks and benefits of wearing CLs before purchasing them. With poor access to the internet and public health education, teenagers living in rural areas are at a high risk of getting ocular complications due to CL wear. Therefore, this study investigates the patterns of use and level of knowledge about CL wear in teenagers residing in rural areas in the state of Selangor, Malaysia.

## 2. Materials and Methods 

This cross-sectional study was carried out from April to December 2014. Sets of validated questionnaires (Supplementary Materials), which were developed by Ky et al. and later translated and validated by Mohidin and Zaimuri, were used in the study [14,15]. The questionnaires consist of 24 questions based on the care and maintenance of CLs, replacement schedules and lens storage cases as well as knowledge about proper CL care and safe usage. The questionnaires were distributed to secondary school students in one period only. The convenience sampling method was used in this study, and the sample size was calculated following Krejcie and Morgan [16]. The number of respondents required was 384 per age group. The total number of subjects needed was 2304.

The study was conducted in five districts (Gombak, Hulu Langat, Hulu Selangor, Klang and Kuala Langat) in Selangor (one of the states in Malaysia). The sampling of schools was done using stratified random sampling from a directory of secondary schools provided by the Ministry of Education (2011). The schools were divided based on districts and locality, into rural and urban categories and the number of students (less than or more than 500) to ensure a sufficient number of respondents. Eight schools were chosen randomly from the rural areas, namely, SMK Tuanku Abdul Rahman, SMK Khir Johari, SMK Sungai Choh, SMK Taman Bunga Raya (1), SMK Pulau Indah, SMK Jenjarom, SMK Sijangkang Jaya and SMK Sungai Manggis.

Prior to data collection, approvals were obtained from the federal government of Malaysia (Ministry of Education Malaysia (KP(BPPDP) 603/5/ JLD.10), the state education office (Education Department of Selangor (JPNS.PPN 600-1/49 JLD.37(34)) and five district education offices, including Gombak (PPDG UPS 6001/39), Hulu Langat (PPDHL.UPPS 100-05/08), Hulu Selangor (PPDHS.PPN 100-12/7/4 Jld. 3(02)), Klang (PPDK/PPS/PPN/04/06/013) and Kuala Langat (PPDKL.PDP 10/4/1 Jld. 2). Ethical approval for the research was obtained from the UKM Research Ethics Committee (UKM 1.5.3.5/244/NN-115-2014), and the protocol followed the tenets of the Declaration of Helsinki in using human subjects for research purposes.

The investigators explained the study to the teachers and school authorities. Furthermore, consent was sought from parents and students prior to data collection. The target participants of the study were students aged 13–18 years in all schools, and the study protocol was explained to them by their teachers. The questionnaires were self-administered and were presented in Bahasa Malaysia (Malaysia’s national language). The students were given one week to complete them. Reminders were given twice by their respective teachers for them to complete the questionnaires. These questionnaires were then collected by their teachers at the end of the week and were later given to the investigators for analysis. The response rate was calculated based on the number of questionnaires distributed and the number of questionnaires collected at the end of the study. The data collected was analysed using SPSS (version 20, IBM, Armonk, NY, USA). Descriptive analysis is used to describe the results.

## 3. Results

A set of 8500 questionnaires were distributed, but only 2474 were completed and returned for analysis. The response rate calculated was 29.1%. The mean age of the respondents was 14.8 ± 1.5 years, ranging from 13 to 18 years in which 60.6% (*n* = 1500) were females and 39.4% (*n* = 974) were males. Following race, majority of the respondents were Malays (*n* = 1784, 72.1%), followed by Chinese (*n* = 418, 16.9%), Indians (*n* = 234, 9.5%) and others (*n* = 38, 1.5%) (Table 1). The age distribution of the respondents is summarized in Table 2.

From the 2474 respondents, 179 (7.2%) of them were CL wearers with 76.0% (*n* = 136) being female. Their reasons for CL wear were for cosmetic purposes (58.1%), comfort (24.6%), therapeutic purposes (7.8%), sports (7.8%) and others (1.7%). Approximately 50.1% of the respondents obtained their lenses from optical shops and optometry practices, whereas 49.9% of them purchased lenses from unlicensed vendors, including beauty accessory shops, followed by friends, the internet and night markets.

From the 179 CL wearers, 92.2% were using soft CLs, and only 7.8% of them were using rigid gas permeable (RGP) lenses. Daily wear soft CLs seemed to be the most preferred modality, given that 93.8% of the respondents were using them. Approximately 75% of the respondents wore their lenses for 8 to 10 h daily, 18.4% for 12 to 18 h daily and 6.1% wore their lenses longer than 18 h daily. Approximately 90% of them claimed to clean and disinfect their CLs using a solution. A multipurpose solution was the most common agent used for lens rinsing after cleaning (45.1%), followed by saline (37.6%) and tap water (17.3%). For disinfecting purposes, approximately 64% of the respondents soaked their CLs for more than six hours, whereas the rest of them performed the procedure in less than four hours. When asked about hand hygiene before lens handling, 85.5% of the respondents indicated that they washed their hands before handling their CLs. As for lens cleaning, 51.3% of the respondents specified that they cleaned their CLs every time before lens insertion, 26.3% did so every time before lens storage, and 10% only performed it sometimes. Approximately 12.5% of the respondents indicated that they cleaned their lenses before lens insertion and lens storage. When asked about the issue of lens sharing, approximately 23% of the respondents admitted having shared their CLs with friends.

The knowledge of CL wear and care were assessed to all respondents, including CL and non-CL wearers. The mean percentage of the correct response amongst CL wearers was 56.5% and 54.41% amongst non-CL wearers. Approximately 67% of the respondents did not know that saline could not be used to disinfect CLs, and 18% of them thought that CLs could be cleaned using tap water. Around 43% of them were not aware that soft CL wearers were at a higher risk of getting corneal infections than RGP lens wearers. The results are summarized in Table 3.

## 4. Discussion

This study illustrates the patterns of use and level of knowledge about CL wear amongst teenagers residing in rural areas in the state of Selangor, Malaysia. The analysis showed a response rate of only 29.1% despite the teenagers being reminded twice by their respective teachers. According to Bowling [17], a low response rate in a questionnaire research indicates unwillingness to participate in the study, the inability of the investigators to contact the respondents and communication barriers (e.g., language). The questionnaires in this study were in Bahasa Malaysia, and the schools were located in Malay-dominated areas, which minimized the communication barriers. Nevertheless, given the time constraints and school regulations, the investigators had limited contact with the students. These factors likely influenced the students’ willingness to participate in the survey. The number of respondents following age was not equally distributed, with the lowest percentage (2.8%) in the 18-year-old group (Table 2). This misdistribution was because the number of 18-year-old students was smaller than the rest of the age groups in the selected schools. In Malaysia, students aged 13 to 17 years undergo secondary education for five years. After secondary education, students can opt to take a preparatory course for one year to pursue higher education in universities. The number of students who choose this option is small, thus influencing the outcome of this study.

The results of this study showed that the prevalence of CL wear amongst teenagers residing in rural areas was 7.2%. However, the low response rate of this study (29.1%) introduced selection bias. This bias would cause a higher prevalence if additional teenage CL wearers participated in the study. To our knowledge, this study is the first to collect a dataset on the prevalence of CL wear amongst teenagers in rural areas in Malaysia. The percentage obtained was lower than the reported prevalence of CL wear amongst teenagers living in urban areas, such as Kuala Lumpur (9.9%) [18] and Rome (9.4%) [19]; such percentage was probably due to the lower prevalence of refractive error and smaller family income. A report from the Department of Statistics, Malaysia (2013) has shown that the average income of families residing in rural areas is lower than the income of those in Kuala Lumpur. The majority of the students possibly could not afford CLs to correct their refractive error given their low family income status. Myopia is the most common refractive error amongst schoolchildren in Malaysia [20,21], but its prevalence is lower in students residing in rural areas than in urban areas [21], thus indicating fewer requirements for optical aids such as CLs for vision correction. Nevertheless, the number of myopic schoolchildren in Malaysia has risen every year due to multiple factors [13,22]. Therefore, the number of teenage CL wearers is projected to increase.

With regard to patterns of use, the results of this study show that CLs are more popular amongst females than males. This finding is similar to that of previous works [18,19] and correlates well with the main purpose of wearing CLs found in this survey, which is for cosmetic use. The majority of the respondents were soft lens wearers, which is also consistent with data from previous studies [18,19]. These results are also consistent with earlier survey findings that show optometrists in Malaysia are more likely to prescribe soft CLs rather than rigid permeable lens (RGP) to their patients due to shorter chair time and initial comfort [10]. The results of this study also shows that daily wear is the popular modality of CL wear amongst the respondents (93.8%), which is also similar to earlier findings [19]. Abbouda et al. reported that approximately 80% of teenage lens wearers in Italy use CLs on a daily basis, 45.7% of which wear lenses for more than nine hours per day. According to the authors, this can be due to the lower price of daily CLs compared with other modalities [19].

Lack of public awareness about the risks of wearing CLs, sharing of CLs and buying CLs from unlicensed vendors are the major concerns amongst eye care practitioners when prescribing CLs to teenagers. The results of this study show that approximately 50% of teenage CL wearers purchase their lenses from unlicensed vendors, such as beauty accessory shops, the internet and night markets. A similar pattern has been observed amongst teenage wearers in urban areas where the majority of them prefer to purchase their CLs from the internet and night markets due to easy access and affordable prices [6]. This purchasing pattern causes apprehension because patients who acquire CLs from unauthorized providers are significantly less likely to acquire appropriate instructions on proper lens use and care and are at risk of eye inflammation and acute vision-threatening infections [23]. These risks are reflected in the results of this study in which a high percentage of wearers have admitted to rinse their lenses using tap water (17%) and share their CLs with friends (22.7%). These inappropriate behaviors may lead to severe visual loss due to exposure to acanthamoeba in tap water and cross-contamination [24].

The final section of the survey in this study consists of six questions about CL wear and care. The results show that only 56% of the respondents answered all the questions correctly, with the lowest percentage (33%) of correct answers about the use of saline to disinfect CLs. Knowing that 18% of the respondents think that CLs can be cleaned using tap water is also worrying. This practice indicates an insufficient level of knowledge, which probably explains why the teenage wearers in this study exhibit non-compliance to CL care. CL wearers younger than 25 years are at an increased risk of corneal inflammatory events (CIEs) during continuous wear of silicone hydrogel CLs, as shown in earlier controlled, randomized, prospective clinical trials and observational studies [23,24]. Even though CIEs do not threaten vision as microbial keratitis does, these events require proper management and may later jeopardize the patient’s ability to continue wearing CLs.

## 5. Conclusions

This study concludes that the prevalence of teenage CL wearers in rural areasin Malaysia is 7.24%. The majority of the CL wearers are females, and the main purpose for wearing CLs is for cosmetic use. Daily soft CL is the most preferred modality, and a multipurpose solution is commonly used to rinse the CLs. The level of knowledge about CL wear and care amongst teenagers is insufficient, thus requiring aggressive ocular public health education to prevent improper behavior during CL wear.

## Figures and Tables

**Table 1:** Demography of respondents.

Respondents	Demography
Total number of respondents (*n*)	2474
Mean age (years)	14.82 ± 1.50
Age range (years)	13 to 18
Gender	Female: 1500 (60.6%) Male: 974 (39.4%)
Race	Malays: 1784 (72.1%) Chinese: 418 (16.9%) Indians: 234 (9.5%)

**Table 2:** Number of respondents following age.

Age (Years)	Number of Respondents
13	579
14	646
15	470
16	269
17	440
18	70
TOTAL	2474

**Table 3:** Summary of results about the patterns of use and knowledge about contact lens wear.

Items	CL Wearers (*n* = 179)	Non-CL Wearers (*n* = 2295)
**Percentage of subjects (%)**	7.2	92
**Reasons for wearing CLs (%)**	Cosmetic (58) Comfort (24.6) Therapeutic (7.8) Sports (7.8) Others (1.7)	
**Type of CLs worn (%)**	Soft CL (92.2) RGP (7.8)	
**Common solutions used for rinsing CLs (%)**	Multipurpose solutions (45.1) saline (37.6) tap water (17.3)	
**Percentage of subjects (%)** **a. Wear CLs more than 12 h/day** **b. Wash their hands before handling CLs** **c. Clean their CLs before storing** **d. Share their CLs with friends**	24.5 85.5 26.3 23	
**Percentage of correct response about CLs (%)**	56.5	54.41

## Supplementary Materials

The following are available online at https://www.mdpi.com/1660-4601/16/24/5161/s1, Research Questionnaire.
