# Supplementary material for: Patterns of Use and Knowledge about Contact Lens Wear amongst Teenagers in Rural Areas in Malaysia
Source: Int J Environ Res Public Health. 2019 Dec 17;16(24):5161. doi: 10.3390/ijerph16245161 (PMC6950730; doi:10.3390/ijerph16245161)
Supplement: Supplementary file 1 [file ijerph-16-05161-s001.pdf]

International Journal of
Environmental Research and Public Health

MDPI

Supplementary Material

Appendix A

RESEARCH QUESTIONNAIRE

TREND DAN PENGETAHUAN TENTANG KANTA SENTUH DI KALANGAN REMAJA SEKOLAH DI KAWASAN LUAR BANDAR DI SELANGOR
(Trend and level of knowledge about contact lens wear amongst teenagers in rural areas in Selangor)

No. Kad Pengenalan: ________________
No. Telefon/Emel: __________________
Tarikh Lahir: ______________________
Jantina: Lelaki Perempuan
Sekolah: __________________________
Tingkatan: ______
Bangsa: Melayu Cina India Lain-lain:______________

Arahan: Sila tandakan √ pada kotak yang bersesuaian.
Jika anda tidak pernah memakai kanta sentuh, sila terus ke soalan 18-24.

1. Berapa lamakah anda telah memakai kanta sentuh?
   Nyatakan ____bulan, ____tahun

2. Dari manakah anda memperolehi kanta sentuh anda?
   - ☐ Rakan
   - ☐ Kedai optikal
   - ☐ Klinik Optometri
   - ☐ Internet
   - ☐ Pasar malam
   - ☐ Kedai aksesori kecantikan
   - ☐ Lain-lain (nyatakan)

   ____

3. Kanta sentuh jenis apakah yang anda pakai?
   - ☐ Kanta sentuh lembut
   - ☐ Kanta sentuh keras

   Sila namakan jenama kanta _____________________________

4. Nyatakan modaliti pemakaian kanta sentuh anda
   - ☐ Pemakaian harian (8-10 jam sehari)
   - ☐ Pemakaian berpanjangan (12-18 jam sehari)
   - ☐ Pemakaian berterusan (selama seminggu)

5. Berikan sebab anda memakai kanta sentuh
   - ☐ Kosmetik
   - ☐ Keselesaan
   - ☐ Terapeutik
   - ☐ Sukan
   - ☐ Lain-lain (nyatakan) _____________

6. Sistem apakah yang anda guna untuk mencuci kanta sentuh anda?
   - ☐ Sistem hidrogen peroksida
   - ☐ Kimia (chemicals)
   - ☐ Pencuci (cleaner)
   - ☐ Disinfektan

- ☐ Salin
- ☐ Tablet enzim
- ☐ Pelbagai guna (multipurpose)
- ☐ Haba
- ☐ Lain-lain (nyatakan) _____________

7. Adakah anda mencuci tangan sebelum mencuci kanta sentuh anda?
    - ☐ Ya
    - ☐ Tidak

8. Adakah anda mencuci kanta sentuh anda?
    - ☐ Ya
    - ☐ Tidak

9. Sekiranya 'ya' bilakah anda mencuci kanta sentuh anda?
    - ☐ Setiap kali menggunakan kanta sentuh
    - ☐ Setiap kali menyimpan kanta sentuh
    - ☐ Kadang-kadang (nyatakan) _______

10. Larutan apakah yang anda gunakan untuk membilas kanta sentuh anda selepas mencucinya
    - ☐ Salin
    - ☐ Pelbagai guna (multipurpose)
    - ☐ Air paip

11. Adakah anda merendam kanta sentuh anda di dalam larutan disinfektan?
    - ☐ Ya
    - ☐ Tidak

12. Berapa lamakah anda merendamnya?
    - ☐ Kurang daripada 4 jam
    - ☐ Lebih daripada 6 jam

13. Adakah anda menggunakan agen rewetting semasa memakai kanta sentuh?
    - ☐ Ya
    - ☐ Kadang-kadang
    - ☐ Tidak

14. Adakah anda menggunakan tablet protein dalam rendaman kanta sentuh anda?
    - ☐ Ya
    - ☐ Tidak

15. Berapa lamakah anda melakukannya?
    - ☐ Kurang daripada 4 jam
    - ☐ Lebih daripada 6 jam

16. Berapa kalikah anda menukar kanta sentuh anda?
    - ☐ Setiap bulan
    - ☐ Setiap tahun
    - ☐ Setiap 2 tahun
    - ☐ Tidak pernah

17. Pernahkah anda berkongsi kanta sentuh dengan rakan anda?
    - ☐ Ya
    - ☐ Tidak

18. Semua jenis kanta sentuh melindungi mata daripada sinaran cahaya ultra lembayung (UV)
    - ☐ Benar

- ☐ Salah

19. Kanta sentuh boleh dibersihkan dengan menggunakan air paip
    - ☐ Benar
    - ☐ Salah

20. Kanta sentuh boleh dipakai semasa berenang sekiranya belodok (goggles) turut dipakai
    - ☐ Benar
    - ☐ Salah

21. Adalah tidak sihat untuk memakai kanta sentuh semasa menggunakan jakuzi
    - ☐ Benar
    - ☐ Salah

22. Pemakai kanta sentuh lembut adalah lebih mudah mendapat jangkitan pada mata berbanding dengan pemakai kanta sentuh keras
    - ☐ Benar
    - ☐ Salah

23. Cecair salin yang steril adalah baik untuk disinfeksi kanta sentuh
    - ☐ Benar
    - ☐ Salah

24. Anda mesti menjalani pemeriksaan mata sekurang-kurangnya sekali setahun selepas pemakaian kanta sentuh
    - ☐ Benar
    - ☐ Salah

Segala jawapan dan maklumat peribadi anda adalah sulit.
Terima kasih di atas kerjasama anda.
